# A newly modified nerve-sparing radical hysterectomy technique with analysis of short-term oncologic, surgical, and functional outcomes

**DOI:** 10.3389/fonc.2025.1555483

**Published:** 2025-06-04

**Authors:** Shichao Han, Xinyou Wang, Jinming Zhu, Ya Li, Jing Na, Jun Wang

**Affiliations:** ^1^ Department of Gynecology and Obstetrics, The Second Affiliated Hospital of Dalian Medical University, Dalian, China; ^2^ Department of Obstetrics and Gynecology, the Second Clinical College of Dalian Medical University, Dalian, China; ^3^ Oncology Department, Affiliated Zhongshan Hospital of Dalian University, Dalian, China

**Keywords:** cervical cancer, radical hysterectomy, nerve-sparing, membrane bridge, embryonic compartment

## Abstract

**Objective:**

This study aims to investigate a newly modified approach to nerve-sparing radical hysterectomy.

**Subjects and Methods:**

Pathological large-section staining of the entire paracolpium from specimens obtained after radical hysterectomy was performed to examine its structural composition and identify the course and distribution of the pelvic plexus and its branches. Based on the analysis of nerve pathways and locations, a total of 55 cases of modified nerve-sparing surgeries preserving pelvic autonomic function were conducted.

**Results:**

All radical hysterectomies were performed in accordance with the principles of membrane anatomy. Postoperative pathological large-section staining of the paracolpium, combined with microscopic examination, revealed three distinct planes from ventral to dorsal: a vascular plane, consisting of the deep uterine vein and bladder venous plexus draining into the deep uterine vein; a neural plane, formed by the pelvic plexus and its nerve branches; and a ligamentous plane, consisting of the sacral ligament. In clinical practice, utilizing the three-plane structure of the paracolpium and medially lifting the vascular plane facilitated safe, reliable dissection, exposure, and preservation of pelvic autonomic nerve function.Through clinical practice, follow-up, and analysis of 55 cases, it was observed that combining the concept of membrane anatomy with the three-plane anatomical structure of the paracolpium not only enables nerve-sparing radical hysterectomy as a bloodless surgical procedure but also ensures optimal nerve preservation, favorable oncological outcomes, and a significant reduction in perioperative complications.

**Conclusion:**

Guided by the principles of membrane anatomy and the three-plane theory of the paracolpium, nerve-sparing radical hysterectomy facilitates precise preservation of pelvic autonomic nerve function in a bloodless manner, while achieving favorable oncological outcomes.

## 1 Introduction

Cervical cancer is the most prevalent gynecological malignancy, with approximately 500,000 new cases diagnosed annually worldwide, the majority of which occur in developing countries (1). Radical hysterectomy remains a cornerstone in the management of early-stage cervical cancer, providing an effective therapeutic option (2). However, this procedure often results in damage to the pelvic autonomic nerves, leading to bladder and rectal dysfunction (3). Complications related to the urinary and gastrointestinal systems can severely impact patients’ quality of life, imposing significant physical and psychological burdens. As a result, nerve-sparing radical hysterectomy (NSRH) has garnered considerable attention as an alternative to traditional radical hysterectomy (RH). NSRH not only minimizes complications associated with the urinary and gastrointestinal systems but also demonstrates no statistically significant differences in 5-year and 10-year disease-free survival (DFS) and overall survival (OS) rates when compared to RH (4). The pelvic nervous system exhibits a complex anatomical structure, the performance of delicate nerve-sparing procedures particularly challenging, comparable to the crown jewel of gynecological surgery.

The total mesometrial resection (TMMR) technique, reported by Höckel et al. (5) and Kim et al. (6), alongside the embryonic compartment-based hysterectomy guided by the membrane anatomy concept described by Han et al. (7–9), suggest that membrane anatomy-based procedures may offer superior oncological outcomes compared to traditional radical surgeries for cervical cancer.

This study aims to comprehensively describe the procedure of embryological compartment-based hysterectomy under the guidance of membrane anatomy principles, with a specific focus on the precise steps involved in preserving the pelvic autonomic nerve branches innervating the bladder.

## 2 Method

### 2.1 Inclusion and exclusion criteria

#### 2.1.1 Inclusion criteria

(1)The tumor is confined to the cervix and the upper segment of the vagina.(2)Imaging studies, including CT, MRI, and PET-CT, show no lymph node metastasis or no distant metastasis.(3)The patient is capable of tolerating the surgical and anesthetic.(4)Staged as IB1 or earlier, including some cases of IB2 and IIA1 with lesions confined to one side of the cervix.(5)The patient has not received radical radiation therapy prior to surgery.

#### 2.1.2 Exclusion criteria

(1)Imaging findings indicate lymph node or distant metastasis.(2)Locally advanced disease (tumor stage IB3 or >IIA1).(3)Previous pelvic radiotherapy or neoadjuvant chemotherapy.(4)Extensive tumor involvement of the parametrium or adjacent structures.(5)The patient is uncapable of tolerating the surgery and Anesthesia.

### 2.2 Patients and specimens

All patients gave their informed consent to the procedure.

### 2.3 Statistical analysis

All data were analyzed using SPSS version 21.0. Given the small sample size and the exploratory nature of this study, only descriptive statistical analyses were conducted on the collected data.

### 2.4 Perioperative considerations

Vaginal flushing: Patients with cervical cancer often experience persistent vaginal bleeding and tumor ulceration, which make the vagina susceptible to inflammation. Preoperative vaginal flushing with povidone-iodine can help maintain cleanliness and reduce congestion in the parametrium and paracolpium due to inflammation. This approach not only facilitates surgical procedures but also helps prevent postoperative infections.

Placement of a D-J tube: In cases of uncertainty regarding ureteral injury during surgery, or if a ureteral injury has occurred, the placement of a D-J (Double-J) stent may be considered to prevent postoperative ureterovaginal fistula.

### 2.5 Surgical stepes

#### 2.5.1 Uterine suspension

**Figure 1:**
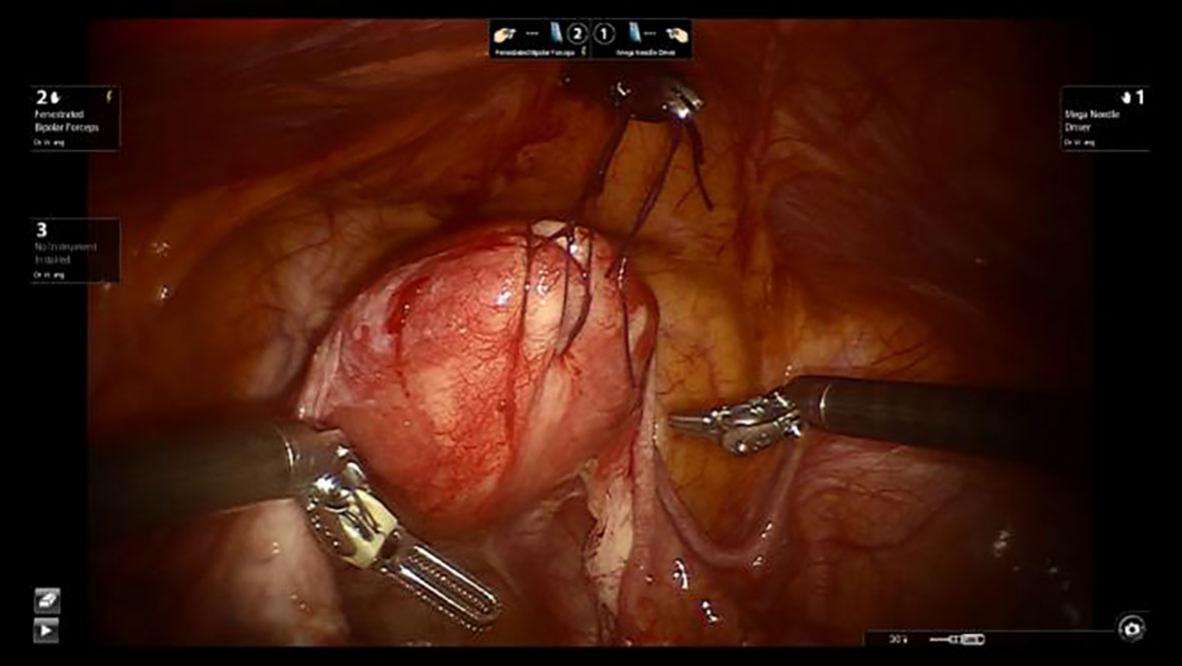
Shows uterine suspension.

Place two stitches at the uterine fundus. A 0.5 cm incision is made above the pubic symphysis, ensuring that the bladder is not injured. A laparoscopic needle holder is inserted to suspend the uterus (see **Figure 1**).

#### 2.5.2 Lateral parametrium

The dissection of the lateral parametrium is critical for preserving pelvic nerve function. Through delicate dissection, the hypogastric plexus and pelvic splanchnic nerves can be identified. In membrane anatomy and the embryonic compartmental theory, the lateral parametrium serves as the mesometrial or “envelope” outlet of the Müllerian embryonic compartment, containing uterine blood vessels and the surrounding lymphatic and adipose tissue. It can also function as a “membrane bridge” (9, 10)connecting the Müllerian embryonic compartment with adjacent compartments. This anatomical structure facilitates material exchange and communication between the Müllerian embryonic compartment, adjacent compartments, and the whole body. Consequently, cervical cancer cells may metastasize to pelvic lymph nodes and even distant organs through the lateral parametrium.

**Figure 2:**
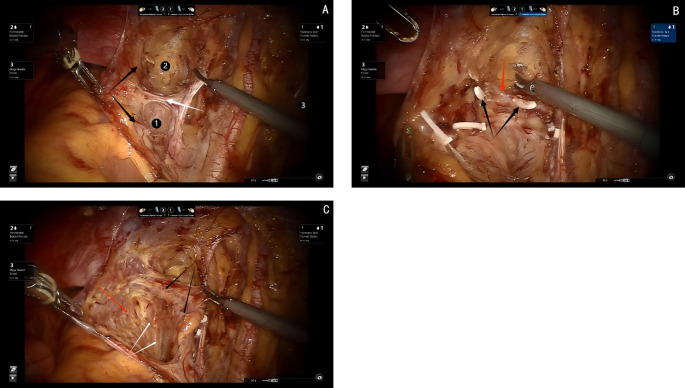
**(A)** black arrows show the ureter, white arrow shows the uterine artery,① shows the Latzko’s prarectal space, ②shows the paravesical space. **(B)** black arrows show the cut end of the deep uterine vein, red arrow shows the pelvic splanchnic pelvic nerves. **(C)** black arrows show the ureter, red arrow shows the Okabayashi’s pararectal space, white arrows show the hypogastric plexus.

The first step in the dissection of the lateral parametrium is to open the lateral peritoneum. During the dissection of the lateral retroperitoneal space, it becomes evident that the anatomy of the lateral parametrium involves the separation of the Müllerian embryonic compartment, ureteric embryonic compartment, urogenital embryonic compartment, and hindgut embryonic compartment, making the dissection relatively complex. However, following the principles of membrane anatomy, each step is performed within avascular embryonic compartmental membrane spaces, allowing for a bloodless surgical procedure. The separation of embryonic compartments in the lateral parametrium can be achieved through precise dissection of Latzko’s pararectal space, the paravesical space, and Okabayashi’s pararectal space. These separations exemplify the concept of embryonic compartmentalization in membrane anatomy. The paravesical space primarily involves the separation between the posterior wall of the bladder from the urogenital embryonic compartment and the mesometrial/envelope outlet of the Müllerian embryonic compartment. Okabayashi’s pararectal space refers to the separation between the ureter and its mesentery from the ureteric embryonic compartment and the rectal mesentery from the hindgut embryonic compartment. Latzko’s pararectal space represents the separation between the mesometrial/envelope outlet of the Müllerian embryonic compartment and the ureter and its mesentery from the ureteric embryonic compartment (see **Figure 2**).

#### 2.5.3 Dorsal parametrium

The dorsal parametrium, also known as the uterosacral ligament, serves as the primary supportive structure of the Müllerian embryonic compartment. This robust ligamentous structure is crucial for maintaining the stability of the Müllerian compartment within the pelvic cavity. Exposure of the dorsal parametrium involves not only the uterosacral ligament but also the posterior vaginal wall. The procedure requires the separation of the uterosacral ligament and the posterior vaginal wall of the Müllerian embryonic compartment from the anterior and lateral mesentery of the hindgut embryonic compartment.

**Figure 3:**
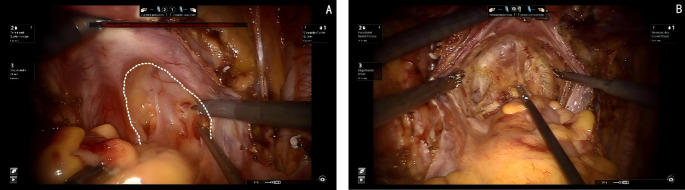
**(A)** Dashed line shows the membrane bridge (know as the rectovaginal fold) between the Müllerian embryonic compartment and the hindgut embryonic compartment. **(B)** Shows the separation of the Müllerian embryonic compartment and the hindgut embryonic compartment.

Since this dissection involves only two embryonic compartments, the procedure is relatively straightforward. The technique begins with transection of the membrane bridge (know as the rectovaginal fold) between the Müllerian embryonic compartment and the hindgut embryonic compartment, providing access to the avascular inter-compartmental space and allowing for a bloodless separation. During the dissection, the dorsal aspect of the uterosacral ligament, where it attaches to the sacral fascia, is visualized. Inferiorly, the ligament adheres closely to the fascia of the levator ani muscle. By carefully detaching the uterosacral ligament from both the sacral fascia and the fascia of the levator ani muscle, the dissection of the dorsal parametrium is completed (see **Figure 3**).

#### 2.5.4 Ventral parametrium

The dissection of the ventral parametrium is the most complex and challenging steps in radical hysterectomy. Accurate dissection of this area is crucial for the successful preservation of pelvic autonomic nerve function. The anatomy in this region involves the separation of the parametrium and paracolpium tissues of the Müllerian embryonic compartment, the ureter of the ureteric embryonic compartment, and the bladder of the urogenital embryonic compartment, which makes the procedures quite difficult.

**Figure 4:**
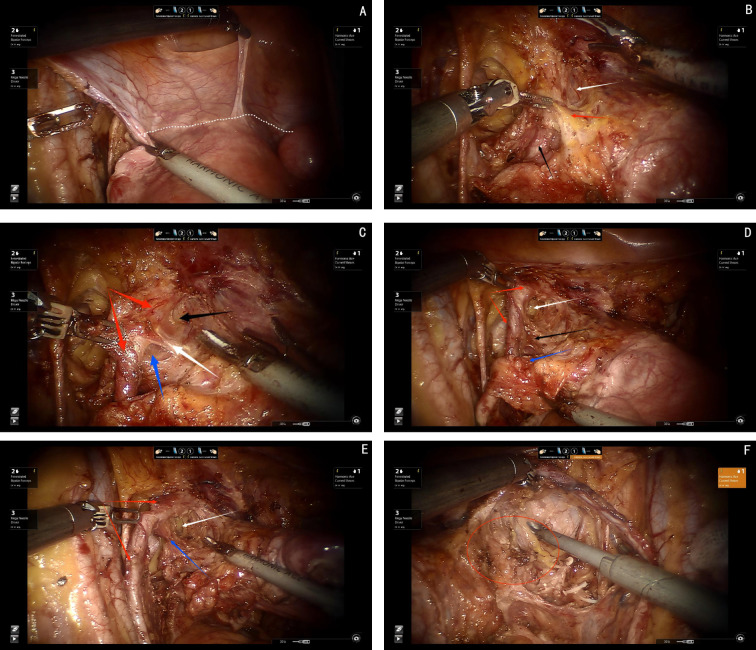
**(A)** Dashed white line shows the membrane bridge between Müllerian embryonic compartment and the urogenital embryonic compartment (i.e., the vesicouterine fold). **(B)** White arrow shows the fourth space, black arrow shows the ureter, red arrow shows the membrane bridge between urogenital and Müllerian embryonic compartments (i.e., the vesicocervical ligament). **(C)** Red arrows show the ureter, black arrow shows the fourth space, blue arrow shows the paravaginal space, white arrow shows the membrane bridge between Müllerian embryonic compartment and the ureteric embryonic compartment. **(D)** Red arrows show the ureter, white arrow shows the paravaginal space, blue arrow shows the uterine artery, black arrow shows vesicovaginal ligament, **(E)** Red arrows show the ureter, white arrow shows the paravaginal space, blue arrow shows the membrane bridge between the urogenital and Müllerian embryonic compartments (i.e., the vesicovaginal ligament), **(F)** Red round circle shows the paracolpium.

First transection of the membrane bridge between the Müllerian embryonic compartment and the urogenital embryonic compartment (i.e., the vesicouterine fold). This allows access to the avascular compartmental spaces between these two compartments, specifically the vesicocervical space and the vesicovaginal space. By continuing to expand the vesicovaginal space laterally, the interstitial space between the intramural segment of the ureter and the parametrium, paracolpium is exposed, which is referred to as Yabuki’s fourth space. Reveal the entire extraperitoneal membrane bridge between the urogenital and Müllerian embryonic compartments (i.e., the vesicocervical ligament). After transecting this membrane bridge, the fibrous connective tissue between the ureter and the parametrium, paracolpium is identified, which know as the membrane bridge between the Müllerian embryonic compartment and the ureteric embryonic compartment. After this membrane bridge is severed, the ureter can be released from the parametrium and paracolpium. Finally, separating the posterior wall of the bladder from the paracolpium of the Müllerian embryonic compartment by exposing of the paravaginal space, allowing for visualization of the last extraperitoneal membrane bridge between the urogenital and Müllerian embryonic compartments (i.e., the vesicovaginal ligament). The transection of this membrane bridge completes the dissection of the ventral parametrium, effectively isolating the Müllerian embryonic compartment (see **Figure 4**).

#### 2.5.5 Nerve-sparing

Preserving the pelvic autonomic nerves, specifically the bladder branches of the inferior hypogastric or pelvic plexus, is an extremely complex surgical procedure, often referred to as the “crown jewel” of gynecologic surgeries. Successfully performing this procedure requires a precise understanding of pelvic nerve anatomy. The pelvic splanchnic nerve plexus is located dorsally to the deep uterine vein in the lateral parametrium, while the hypogastric nerve bundle lies dorsally to the ureteral mesentery, running laterally along the uterosacral ligament of the dorsal parametrium. Ultimately, the two types of nerves converge dorsolaterally in the paracolpium, forming the inferior hypogastric plexus or pelvic plexus. This plexus gives rise to nerve branches that innervate the uterus, cervix, vagina, bladder, and rectum, creating a well-defined neural plane.

The vesicovaginal ligament houses the bladder venous plexus, which drains into the deep uterine vein. The deep uterine vein is located ventrally to the pelvic splanchnic nerve plexus, meaning the vascular plane formed by the bladder venous plexus and the deep uterine vein lies ventrally to the neural plane. The hypogastric nerve bundle courses laterally along the uterosacral ligament of the dorsal parametrium, making the posterior aspect of the neural plane correspond to the dorsal ligament plane. The paracolpium consists of the vesicovaginal ligament in the ventral parametrium, the deep uterine vein and its surrounding lymphatic-adipose tissue in the lateral parametrium, and the uterosacral ligament in the dorsal parametrium. Consequently, the paracolpium is divided into the ventral vascular plane, the middle neural plane, and the dorsal ligamentous plane.

With this precise anatomical understanding of the paracolpium, surgeons can effectively preserve the bladder branches of the inferior hypogastric plexus/pelvic plexus, ultimately achieving the “crown jewel” of nerve-sparing surgery.

**Figure 5:**
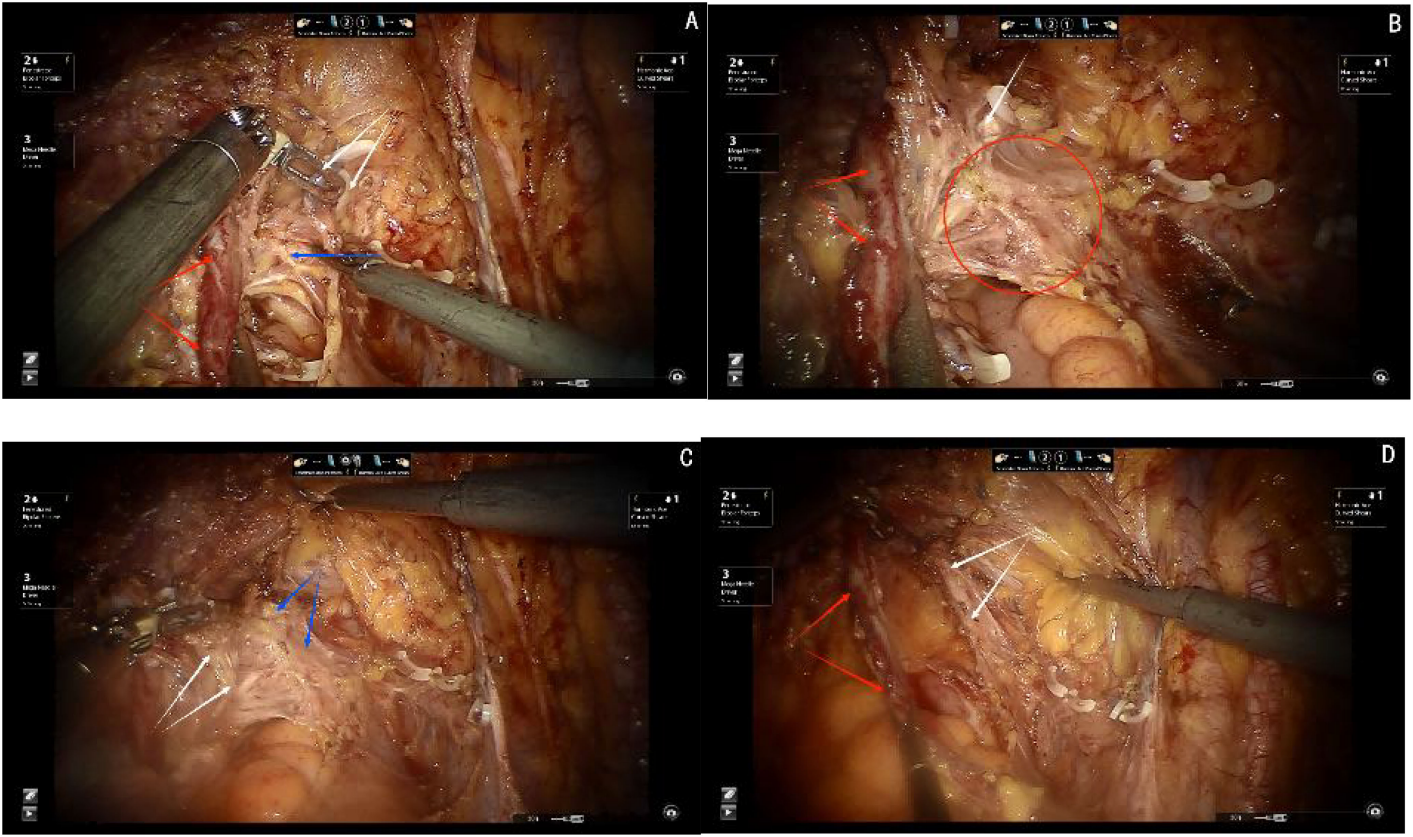
**(A)** Red arrows show the ureter, blue arrow shows the inferior hypogastric plexus(IHP), white arrows show the cut ends of deep uterine vein and bladder venous. **(B)** White arrows show the venous plane, red arrows show the ureter, red circle show the neural plane(IHP and branches of IHP). **(C)** White arrows show the cut ends of the IHP branches, black arrows show the ligamentous palne, blue arrows shows the bladder branches of IHP. **(D)** Red arrows show the ureter, white arrows show the bladder branches of IHP.

Step one, elevating the deep uterine vein and the bladder venous plexus, which runs within the vesicovaginal ligament and drains into the deep uterine vein, as a single vascular plane toward the midline. Sharp dissection is then performed to expose the inferior hypogastric plexus/pelvic plexus and its nerve branches (see **Figures 5A, B**).

Step two, transection of all nerve branches, except the bladder branch, allowing for the clear visualization of the ligamentous plane dorsal to the neural plane. For improved surgical outcomes and clearer visualization, particularly for broader academic understanding, the Fujii space can be exposed. In this step, the bladder branch of the pelvic plexus/inferior hypogastric plexus is separated from the medial nerve branches, transforming the cross-shaped structure of the pelvic plexus/inferior hypogastric plexus from a “+” or “X” configuration to a “Y” shape (see **Figure 5C**).

Step three, transection of the ligamentous plane vertically, perpendicular to the vagina, along the fascia of the levator ani muscle (see **Figure 5D**).

**Figure 6:**
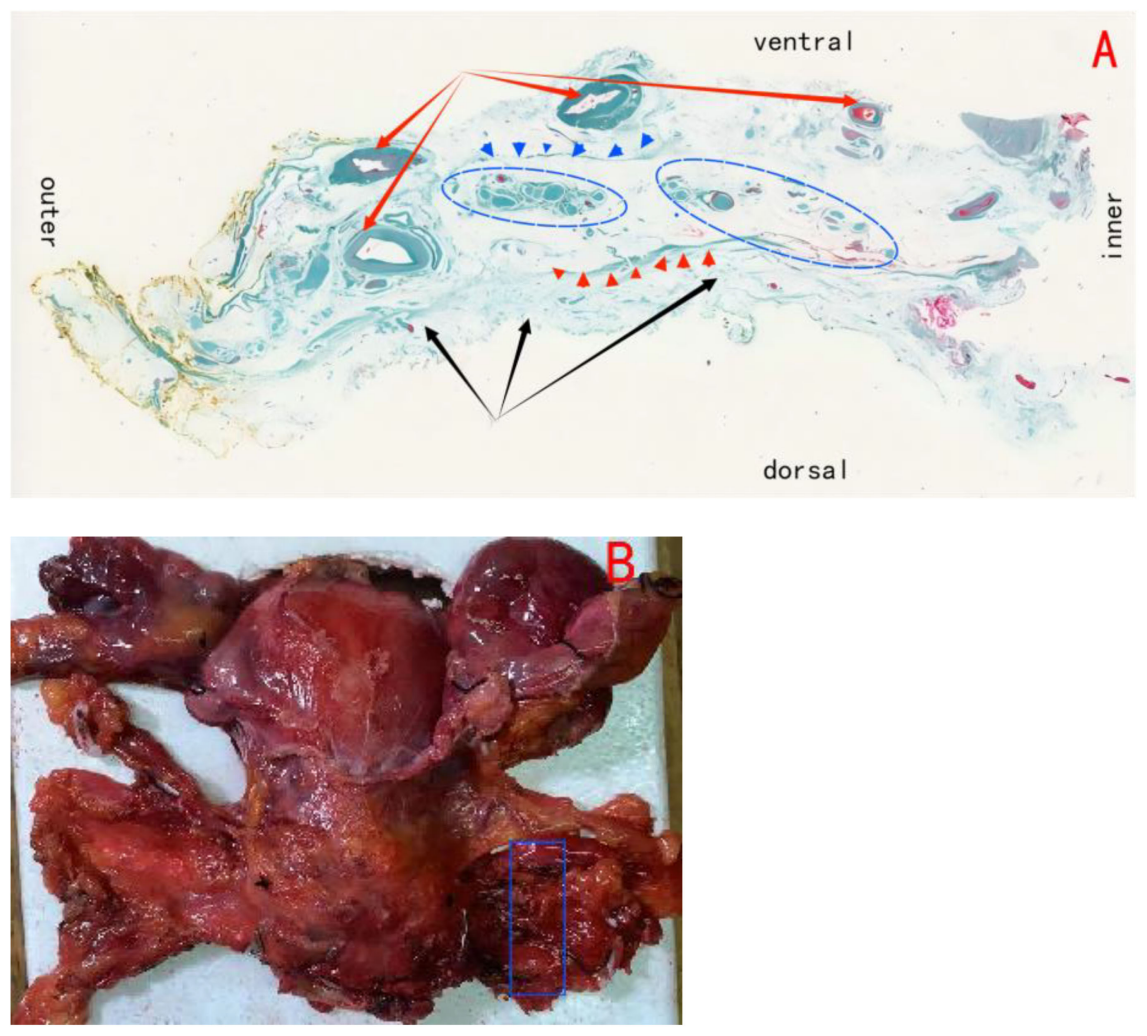
**(A)** Shows the paracolpium; black arrows show the ligamentous plane(i.e., the utrosacral ligament); red arrows show the vascular plane, composed of the deep uterine vein and the bladder venous plexus draining into the deep uterine vein; blue circles show the neural plane, formed by the pelvic plexus and its nerve branches; blue triangles show the border line between vascular plane and neural plane; red triangles show the border line between neural plane and ligamentous plane. **(B)** Blue area shows the **(A)**.

We can observe the pathological analysis of paracolpium as illustrated in **Figure 6**.

#### 2.5.6 Postoperative complication evaluation

##### 2.5.6.1 Gastrointestinal complications

Intestinal Obstruction: Persistent need for gastrointestinal decompression beyond 7 days postoperatively or radiographic evidence of bowel dilation (excluding early postoperative paralytic ileus).

Enterocutaneous Fistula: Contrast imaging/CT demonstrating leakage of intestinal contents or drainage of intestinal fluid through a drainage tube.

Rectal Dysfunction: New onset constipation or incontinence postoperatively.

##### 2.5.6.2 Urinary system complications

Urinary Retention: Residual urine volume >150 mL post-catheter removal (measured by ultrasound).

Urinary Tract Infection: Positive urine culture with symptoms or pyuria.

##### 2.5.6.3 Vascular complications

Intraoperative Bleeding: Blood loss ≥800 mL or requirement for blood transfusion (WHO classification).

Deep Venous Thrombosis: Confirmation of thrombus by ultrasound/CT with clinical symptoms.

## 3 Results

A total of 55 patients with cervical cancer who underwent surgical treatment of the QM-C1 type were included in the study. All patients received an embryonic compartment-based hysterectomy. Pelvic autonomic nerve function preservation was performed using the three-plane theory of the paracolpium (which consists of the vascular plane, neural plane, and ligament plane from ventral to dorsal). The median age of all included patients was 52 (range 28–72) years. Based on the FIGO 2018 staging, the preoperative staging of the patients was assessed as follows: IA2–1 case (1.81%), IB1–45 cases (81.82%), IB2–9 cases (16.36%). Seven (12.73%) had adenocarcinoma and 48 (87.27%) had squamous cell carcinoma. Ten (18.18%) patients had G1 tumors and 45 (81.82%) had G2 tumors (**Table 1**).

**Table 1:** Patients’ clinical characteristics.

Characteristics	All Patients n = 55 (%)
mean age	52.11 (28-72)
Smoking	
Yes	1 (1.82)
No	54 (98.18)
HPV type	
16, 18	47 (85.5)
Other	7 (12.7)
mean SCC (ng/ml)	0.62 (2.52-14.47)
mean Hb change (g/l)	8.81 (15 - 33)
FIGO stage	
IA2	1 (1.81)
IB1	45 (81.82)
IB2	9 (16.36)
Histology	
Squamous cell carcinoma	48 (87.27)
Adenocarcinoma	7 (12.73)
Lymphovascular space invasion(LVSI)	
Yes	16 (29.09)
No	39 (70.91)
Grading	
G1	10 (18.18)
G2	45 (81.82)
G3	0

The surgical approaches included 10 cases (18.18%) of laparotomy, 36 cases (65.45%) of laparoscopy, and 9 cases (16.36%) of robot-assisted laparoscopy. There were no cases of conversion to laparotomy. Fifty-two patients underwent pelvic lymph node dissection, and 3 patients underwent pelvic and abdominal aortic lymph node dissection. The median number of pelvic lymph nodes removed was 23 (range 8–48). Three patients had pelvic lymph node metastasis, no patient had para-aortic lymph node metastasis, and 29.09% of cases had lymphovascular space invasion. The resection margins were negative in all cases.

No tumor rupture or fragmentation occurred during any of these QM-C1 surgeries. R0 resection was achieved in all 55 patients (100%). The median operating time was 282 minutes (range 160–496 minutes), and the estimated median blood loss (EBL) was 72 mL (range 5–400 mL). No patients required intraoperative or postoperative transfusions. The median postoperative hemoglobin change was 8.81 g/dL (range -15 to 33 g/dL) (**Table 2**).

**Table 2:** Operation characteristics.

Characteristics	All Patients n = 55 (%)
mean operation time (min)	282.64 (160-496)
mean bleeding (ml)	72.27 (5-400)
Approach	
open	10 (18.18)
laparoscopy	36 (65.45)
robot	9 (16.36)
mean ureteral placement duration (week)	1.51 (1-2.40)
Recurrence	
Yes	0
No	55 (100)
mean Follow-up (month)	15.38 (2-44)*
Complications	
Yes	1 Delayed wound healing
No	54 (100)
Spontaneous urination after catheter removal	
Yes	55 (100)
No	0
Postoperative satisfactory defecation	
Yes	55 (100)
No	0
Recurrence	
Yes	0
No	55 (100)
Adjuvant treatment	
Yes	0
No	55 (100)

No intraoperative or postoperative complications such as vascular, intestinal, ureteral or bladder injuries were observed. Only one patient experienced postoperative fat liquefaction. All patients completed the procedure without adjuvant therapy, with a median duration of catheter placement of 3 weeks (range 1–4 weeks), and all 55 patients achieved autonomous urination and urinary continence after catheter removal. No postoperative intestinal dysfunction was reported.

No patients were treated with adjuvant therapy after surgery. No local or distant recurrence or metastasis was found during the median follow-up of 15 months.

## 4 Discussion

Currently, for the surgical treatment of cervical cancer, guidelines recommend adhering to the “A-B-C-D” four-type classification (Q-M classification) proposed by Querleu and Morrow in 2008 (11). In 2017, Querleu et al. updated the Q-M classification, suggesting that type C1 surgery should be the primary option for type C cases, while type C2 surgery should only be considered when autonomic nerve preservation is not feasible due to anatomical constraints (12). Improving postoperative pelvic organ function and enhancing patients’ quality of life are the core objectives of C1 surgery. However, isolating the complex autonomic structures in the paracervical connective tissue is challenging, and even experienced surgeons may struggle to ensure precise nerve preservation during surgery. Therefore, simplifying C1-type surgery is of particular importance. A comprehensive understanding of pelvic organ and autonomic nerve anatomy is fundamental to performing standardized procedures (13). Pelvic nerve anatomy, particularly the precise localization of the plexus and its branches, has been a primary focus of research.

The study of pelvic nerve anatomy, particularly the precise localization of the pelvic plexus and its branches, has remained a central focus of research. Preserving autonomic nerve function during radical hysterectomy is an exceptionally challenging task, and even highly experienced surgeons may find it difficult to achieve precise nerve preservation in such procedures.

Through pathological analysis of large sections of the paracolpium, our team identified that this tissue consists of a “sandwich-like” composite structure, comprising vascular, neural, and ligamentous planes. By applying the three-plane theory of the paracolpium during radical hysterectomy, the vascular plane can be lifted medially, allowing for bloodless and precise exposure of the pelvic plexus and its branches. This technique facilitates the accurate preservation of autonomic nerve function in the pelvis.

Using this three-plane theoretical system, we performed autonomic nerve-preserving surgeries on 55 patients, successfully achieving precise nerve preservation. During a follow-up period of up to 44 months, none of the patients experienced local or distant oncological recurrence. Furthermore, all patients avoided postoperative bowel dysfunction. The average time to catheter removal was 1.51 weeks, which is consistent with durations reported in other studies (4). After catheter removal, all patients regained autonomous urination and urinary continence. These findings demonstrate that the technique is both safe and reliable.

Among the 55 cases, 44 were performed using minimally invasive approaches: 36 via laparoscopy, 9 via robotic surgery, and 10 via open surgery. Although the sample size and follow-up period were limited, the results suggest that different surgical approaches had minimal impact on the preservation of pelvic nerve function. However, further validation of oncological outcomes across various surgical approaches will require additional cases and multicenter randomized controlled trials.

Based on an in-depth understanding of traditional anatomy, our team proposed and performed total Müllerian compartment resection(TMCR) (10) under the concept of membrane anatomy, combining the concept of embryonic development. The compartment-based resection approach, grounded in membrane anatomy and embryonic development theory, redefines the principles of radical tumor surgery. Rather than primarily focusing on the margin width from the tumor to the specimen edges, this strategy emphasizes the complete excision of the entire embryonic compartment as the key standard. By prioritizing the thorough removal of the compartment, this method enhances local tumor control, often eliminating the need for adjuvant radiation. Simultaneously, it minimizes complications and preserves the functional integrity of adjacent embryonic compartments.

Resection strategies based on embryonic compartments can be tailored to include extensive resections within a single compartment or expanded to supra-compartmental or multi-compartmental approaches, depending on the tumor stage. For advanced local malignancies, these expanded techniques provide viable treatment options. Clinical studies in cervical cancer (14, 15), vulvar cancer (16), vaginal cancer (17), and rectal cancer [19] have demonstrated the efficacy of this approach. These findings highlight the potential of compartment-based resection to improve local tumor control, reduce treatment-related complications, and enhance overall survival outcomes.

Professor Höckel’s TMMR surgery has shown significant advantages in oncological outcomes and perioperative complications. However, this technique does not clearly define the boundaries between the mesenteries of various organs, making it difficult to replicate. This may be one of the primary reasons why the technique, despite being developed for over 20 years, has not been widely accepted or implemented. Our team’s TMCR technique proposes that the membrane bridges serve as the connection and boundary between embryonic compartments (18). These membrane bridges represent the boundaries between the embryonic compartments, making the technique easier to understand, master, and apply widely in clinical practice. TMCR allows for safe embryonic compartment resection by severing the membrane bridges, which also aligns with the tumor-free principle of avoiding extravasation of tumor cells and ensuring complete resection of the embryonic compartment integrity.

The nerve-sparing radical hysterectomy is a classic embryonic compartment-based surgical approach. When combined with the principles of membrane anatomy, it shows potential for achieving favorable oncological outcomes. However, this study also has limitations that need to be addressed in future research. First, this study did not include a control group. The primary reason is that once the TMCR is performed, achieving precise, bloodless nerve-sparing tumor resection, it becomes difficult to revert to traditional surgery, thus no control group was set. In the future, we can incorporate a multicenter study to include a control group for comparison. Secondly, the follow-up period for this study is relatively short. The short-term follow-up results of this technique are indeed promising, and the team wanted to share this technique with others. However, regarding the technical innovation in tumor surgery, the oncological safety of the procedure still needs to be monitored. Therefore, the team is accumulating data and plans to publish the 3-year or 5-year OS and DFS of patients in the future to demonstrate the advantages and disadvantages of this technique and verify whether it is practical.

## 5 Conclusion

This study is the first to present pelvic autonomic nerve-sparing techniques to the global community using a novel concept, an intuitive approach, and detailed procedural steps. However, it is limited by a small sample size and a short follow-up duration. To overcome these limitations, further studies with larger sample sizes and longer follow-up periods are needed to validate the safety of this technique.

## Data Availability

The original contributions presented in the study are included in the article/supplementary material. Further inquiries can be directed to the corresponding author.

## References

[1] Hoque MR Haque E Karim MR. Cervical cancer in low-income countries: a Bangladeshi perspective. Int J Gynaecol Obstet. (2021) 152:19–25. doi: 10.1002/ijgo.v152.1 32989750

[2] Davidson T Yakobi Y Nayruz K Levin G Korach J Perri T. Early prediction of urinary retention following radical hysterectomy by 18FFDG PET/CT imaging. Minerva Obstet Gynecol. (2023) 75:243–50. doi: 10.23736/S2724-606X.21.05008-9 34904588

[3] Cibula D Velechovska P Slama J Fischerova D Pinkavova I Pavlista D. Late morbidity following nerve-sparing radical hysterectomy. Gynecol Oncol. (2010) 116:506–11. doi: 10.1016/j.ygyno.2009.10.061 19906412

[4] Yamamoto A Kamoi S Ikeda M Yamada T Yoneyama K Takeshita T. Effectiveness and long-term outcomes of nerve-sparing radical hysterectomy for cervical cancer. J Nippon Med Sch. (2021) 88:386–97. doi: 10.1272/jnms.JNMS.2021_88-503 32741908

[5] Falconer H Norberg-Hardie A Salehi S Alfonzo E Weydandt L Dornhöfer N. Oncologic outcomes after Total Mesometrial Resection (TMMR) or treatment according to current international guidelines in FIGO (2009) stages IB1-IIB cervical cancer: an observational cohort study. EClinicalMedicine. (2024) 73:102696. doi: 10.1016/j.eclinm.2024.102696 39007068 PMC11245980

[6] Kimmig R Wimberger P Buderath P Aktas B Iannaccone A Heubner M. Definition of compartment-based radical surgery in uterine cancer: radical hysterectomy in cervical cancer as 'total mesometrial resection (TMMR)' by M Höckel translated to robotic surgery (rTMMR). World J Surg Oncol. (2013) 11:211. doi: 10.1186/1477-7819-11-211 23972128 PMC3765976

[7] Na J Li Y Wang X Song S Wang J Han S. Clinical application of embryonic compartment hysterectomy in the treatment of cervical cancer. Chin J Pract Gynecology Obstetrics. (2024) 40:209–14. doi: 10.19538/j.fk2024020116

[8] Han S Na J Li Y Wang J. Surgical procedures and techniques in robot-assisted uterine artery-preserving radical trachelectomy. J Robot Surg. (2024) 18:222. doi: 10.1007/s11701-024-01982-y 38795189

[9] Han S Na J Li Y Lu J Wang J. Application and prospect of membrane anatomy in robot-assisted laparoscopic cervical cancer surgery. J Robotic Surg. (2023) 4:456–63. doi: 10.12180/j.issn.2096-7721.2023.05.009

[10] Höckel M Horn LC Fritsch H. Association between the mesenchymal compartment of uterovaginal organogenesis and local tumour spread in stage IB-IIB cervical carcinoma: a prospective study. Lancet Oncol. (2005) 6:751–6. doi: 10.1016/S1470-2045(05)70324-7 16198980

[11] Querleu D Morrow CP. Classification of radical hysterectomy. Lancet Oncol. (2008) 9:297–303. doi: 10.1016/S1470-2045(08)70074-3 18308255

[12] Querleu D Cibula D Abu-Rustum NR. 2017 Update on the querleu–Morrow classification of radical hysterectomy. Ann Surg Oncol. (2017) 24:3406–12. doi: 10.1245/s10434-017-6031-z PMC6093205 28785898

[13] Yang Y Qin T Zhang W Wu Q Yang A Xu F. Laparoscopic nerve-sparing radical hysterectomy for bulky cervical cancer (≥6 cm) after neoadjuvant chemotherapy: A multicenter prospective cohort study. Int J Surg. (2016) 34:35–40. doi: 10.1016/j.ijsu.2016.08.001 27519498

[14] Höckel M Horn LC Manthey N Braumann UD Wolf U Teichmann G. Resection of the embryologically defined uterovaginal (Müllerian) compartment and pelvic control in patients with cervical cancer: a prospective analysis. Lancet Oncol. (2009) 10:683–92. doi: 10.1016/S1470-2045(09)70100-7 19482513

[15] Höckel M Schmidt K Bornmann K Horn LC Dornhöfer N. Vulvar field resection: Novel approach to the surgical treatment of vulvar cancer based on ontogenetic anatomy. Gynecol Oncol. (2010) 119:106–13. doi: 10.1016/j.ygyno.2010.06.019 20650508

[16] Höckel M Horn LC Illig R Dornhöfer N Fritsch H. Ontogenetic anatomy of the distal vagina: Relevance for local tumor spread and implications for cancer surgery. Gynecol Oncol. (2011) 122:313–8. doi: 10.1016/j.ygyno.2011.04.040 21621829

[17] Höckel M Horn LC Hentschel B Höckel S Naumann G. Total mesometrial resection: high resolution nerve-sparing radical hysterectomy based on developmentally defined surgical anatomy. Int J Gynecol Cancer. (2003) 13:791–803. doi: 10.1136/ijgc-00009577-200311000-00010 14675316

[18] Li Y Na J Wang X Han S and Wang J. Robot-assisted Müllerian compartment resection for cervical cancer. Front Oncol. (2024) 14:1466921. doi: 10.3389/fonc.2024.1466921 39474108 PMC11519682

